# The Establishment of Metabolic Syndrome Model by Induction of Fructose Drinking Water in Male Wistar Rats

**DOI:** 10.1155/2014/263897

**Published:** 2014-06-18

**Authors:** Norshalizah Mamikutty, Zar Chi Thent, Shaiful Ridzwan Sapri, Natasya Nadia Sahruddin, Mohd Rafizul Mohd Yusof, Farihah Haji Suhaimi

**Affiliations:** ^1^Anatomy Discipline, Surgical Science Cluster, Medical Faculty, Universiti Teknologi MARA, Sungai Buloh Campus, Jalan Hospital, 47000 Sungai Buloh, Selangor, Malaysia; ^2^Anatomy Department, Medical Faculty, Universiti Kebangsaan Malaysia, Jalan Raja Muda Abdul Aziz, 50300 Kuala Lumpur, Malaysia

## Abstract

*Background*. Metabolic syndrome can be caused by modification of diet by means of consumption of high carbohydrate and high fat diet such as fructose. *Aims*. To develop a metabolic syndrome rat model by induction of fructose drinking water (FDW) in male Wistar rats. *Methods*. Eighteen male Wistar rats were fed with FDW 20% and FDW 25% for a duration of eight weeks. The physiological changes with regard to food and fluid intake, as well as calorie intake, were measured. The metabolic changes such as obesity, dyslipidaemia, hypertension, and hyperglycaemia were determined. Data was presented in mean ± SEM subjected to one-way ANOVA. *Results*. Male Wistar rats fed with FDW 20% for eight weeks developed significant higher obesity parameters compared to those fed with FDW 25%. There was hypertrophy of adipocytes in F20 and F25. There were also systolic hypertension, hypertriglyceridemia, and hyperglycemia in both groups. *Conclusion*. We conclude that the metabolic syndrome rat model is best established with the induction of FDW 20% for eight weeks. This was evident in the form of higher obesity parameter which caused the development of the metabolic syndrome.

## 1. Introduction

Metabolic syndrome encompasses cluster of risk factors for cardiovascular disease which include abdominal obesity, dyslipidemia, hypertension, and hyperglycemia [1]. The incidence of metabolic syndrome is on the rise globally, thereby leading to an increase in the prevalence of metabolic syndrome [2]. Statistics reveal 20–25% of adult population in the world being diagnosed as metabolic syndrome [3].

Factors that contribute to the development of metabolic syndrome are high carbohydrate and high fat diet as well as sedentary lifestyles. All these factors are reversible. Thus, emphasis on the diet modification and exercise are always advised in conjunction with drug intervention.

Prevention of metabolic syndrome is important as the complication of cardiovascular disease increased 2-fold from five to ten years compared to the normal patients [2]. The mortality due to cardiovascular complications is increased when a person has more than one component in the metabolic syndrome [4]. Meanwhile, the risk of getting diabetes has increased 5-fold in the metabolic syndrome patients [2].

Various definitions of metabolic syndrome have been coined by various organizations such as World Health Organization (WHO), International Diabetes Federation (IDF), and National Cholesterol of Adult Treatment Panel III (NCEP ATP III) [2]. Later, these organizations combined and developed a new definition of metabolic syndrome known as “harmonized criteria” which included central obesity, raised blood pressure, elevated triglyceride levels, low high-density lipoprotein (HDL), and raised glucose levels [2].

Fructose is a simple monosaccharide that has been used as a sweetener in food and drinks [5]. On an average, the consumption of fructose has increased to 16% from 1986 to 2007 [6]. The increase in consumption of fructose is closely related to the incidence of obesity [7]. One of the reasons why fructose causes obesity is due to fructose not being able to stimulate the secretion of insulin from pancreatic *β*-cells. This is due to an absence of GLUT5 transporter from the pancreatic *β*-cell [7]. Furthermore, the metabolism bypasses the main pathway of glycolysis which converts glucose-6-phosphatase to fructose-1, 6-biphosphate by phosphofructokinase enzyme [8]. These two factors counteracted with the glucose that stimulates the secretion of insulin from pancreatic *β*-cell which converts the glucose to glycogen. The metabolism of glucose also undergoes the rate limiting step in glycolysis pathway.

There are two types of rats, namely, Wistar rats and Sprague-Dawley (SD) rats, which have been commonly used in the study of metabolic disease [9]. These rats are able to show the increase in body weight, triglyceride (TG) level, and hypertension. However, Wistar rats are more active compared to SD rats. Earlier studies compared the metabolic effect between Wistar and SD rats following consumption of FDW 10% for eight weeks as shown by hypertension and hypertriglyceridemia experimental rats [10]. However, no metabolic changes were observed in the Wistar rats [10]. These differences could be attributed to the active behaviour of Wistar rat leading to a higher metabolic rate.

FDW 20% was used in the study as per an earlier protocol involving SD rats [11]. To date, there is paucity of research which investigated the metabolic effects in Wistar rats with consumption of FDW 20% and FDW 25% even with the consumption of normal diet.

The present study was conducted to develop a metabolic syndrome rat model induced by the FDW even with the consumption of normal diet. To our best of knowledge, the development of metabolic syndrome model is attributed to the modification of diet which include the consumption of high carbohydrate and high fat diet (HCHF) in male Wistar rat [12].

The main aim of this study was to establish a metabolic syndrome rat model by consumption of FDW even with the consumption of normal diet. With the development of a new metabolic syndrome rat model, it may help all researchers in future, especially those involved in the metabolic syndrome niche area whereby this model is easily formed, cost-effective with better time efficiency.

## 2. Materials and Methods

### 2.1. Animals and Diets

Eighteen male Wistar rats with a body weight between 250 and 300 g were obtained from the Animal House of Universiti Kebangsaan Malaysia. The rats were housed in temperature-controlled (20–22°C) room on a 12:12 h dark-light cycle. The rats were acclimatised for 14-day period to the environment with free access to food and water. The experimental protocols were approved by the Animal Ethics Committee of Universiti Kebangsaan Malaysia (FP/ANAT/2012/FARIHAH/18-JULY/453-JULY-2012–AUGUST-2013).

The rats were randomly divided into three groups and fed with standard rat chow (Gold Coin, Sdn. Bhd.) with difference in water consumption for eight weeks. The rats that received normal tap water, fructose 20% in drinking water, and fructose 25% in drinking water were grouped as C, F20, and F25, respectively. Each group consisted of equal number of rats (*n* = 6).

### 2.2. Preparation of Fructose Drinking Water

The fructose that was used was D-fructose >99% (Syarikat System Malaysia). Fructose drinking water was freshly prepared every alternate day [13] and was based on weight/volume formula [14]. To prepare fructose 20% drinking water, 20 g of fructose was diluted in 100 mL of tap water. Meanwhile, for fructose 25% drinking water, 25 g of fructose was diluted in 100 mL of tap water. The bottles were then covered with aluminium foil to prevent fermentation [11]. The FDW was administered every day for eight weeks* ad libitum*.

### 2.3. Physiological Measurements

Daily food intake, fluid intake, and calorie intake were measured every day for eight weeks and the mean was compared. The food and fluid intake for each rat were measured by subtracting the measured amount provided to the remaining amounts in the cage [11]. The calorie intake was calculated based on the amount of food and fluid intake and the corresponding constants [11].

### 2.4. Obesity Parameters

Percentage of body weight gain, body mass index (BMI), and abdominal circumference (AC) were measured as indicators of obesity. Body weight was taken on weekly basis using the electronic weighing scale. The increment of body weight was calculated by subtracting the final weight from the initial weight and the percentage was calculated.

Body mass index and AC were measured twice at baseline and at the end of the experiment. The BMI was calculated by dividing the weight (g) by the length (cm²) [15]. The length of the rats was measured between nasal and anal region [12].

Abdominal circumference was measured using the measuring tape around the anterior abdomen in centimetre [15]. All the measurements were done in anaesthetized rats [12]. The rats were anaesthetized by inhalation of diethyl ether [16].

### 2.5. Blood Pressure Measurements

Blood pressure was measured using the tail-cuff method with sphygmomanometer technique using Power Lab data at the baseline and at the end of the experiment. The rats were anaesthetized by inhalation of diethyl ether before the measurement was taken [17]. Three readings were taken consecutively and the average was then calculated and taken as a final reading for SBP [17].

### 2.6. Blood Biochemistry

Blood samples were attained at the baseline and at end of the experiment via orbital vein in anaesthetized rats. The rats were fasted overnight and supplemented with only tap water [12]. The drinking water in FDW in F20 and F25 groups was replaced with tap water [12]. The plasma samples were sent to lab for analysis of lipid profile and glucose.

### 2.7. Gross and Microscopic Changes of Adipose Tissue

After the rats were sacrificed by using inhalation of diethyl ether [16], a longitudinal incision was given at anterior aspect of the body. The depositions of abdominal adipose tissue which included the omental, retroperitoneal, and epididymal fat were observed* in situ* and then were removed and dapped with gauze before weighing. The weight of adipose tissue was normalized to tibial length and expressed as milligram per millimetre tibial length (mg/mm) [18].

Then, the adipose tissues were immediately fixed in the 10% formalin for three days. These tissue samples were processed. Thin sections (5 *μ*m) were obtained and stained with haematoxylin and eosin (H&E) for histomorphometry of adipocytes. Finally, the sections were mounted on dibutyl phthalate in xylene (DPX). Histomorphometry of adipocytes was analyzed with Video *t*-test Morphology 5.1 software. Three measuring areas 350 *μ*m × 250 *μ*m were calculated in each of the specimens. To count the number of adipocytes cells, each cell in the measuring area was counted and the cells in the border were left out. To measure the size of adipocyte, three measurements including the area, perimeter, and diameter were measured.

### 2.8. Statistical Analysis

All data was analysed with Statistical Package for Social Sciences (SPSS, version 20) and were presented as mean values with their standard error of means (SEM) and subjected to one-way ANOVA with significant *P* value as <0.05.

## 3. Results

### 3.1. Physiological Parameter

Table 1 showed the effect on physiological following consumption of FDW 20% and FDW 25% drink. The food intake was decreased significantly with consumption of FDW 20% and FDW 25% compared to C group. The amount of food consumed by F20 and F25 did not differ. Hence, there was no statistical difference between these groups. On the other hand, the fluid intake was significantly high in the F20 group followed by C group and significantly low in F25 group.

Although the food intake was significantly low with consumption of FDW 20% and 25%, the total calorie intake was significantly high in these groups compared to C group. The value of the total calorie intake was significantly higher in F20 compared to F25 group which reflected the calorie consumption from the drinking water was greater with consumption of FDW 20%.

### 3.2. Obesity Parameter

**Figure 1:**
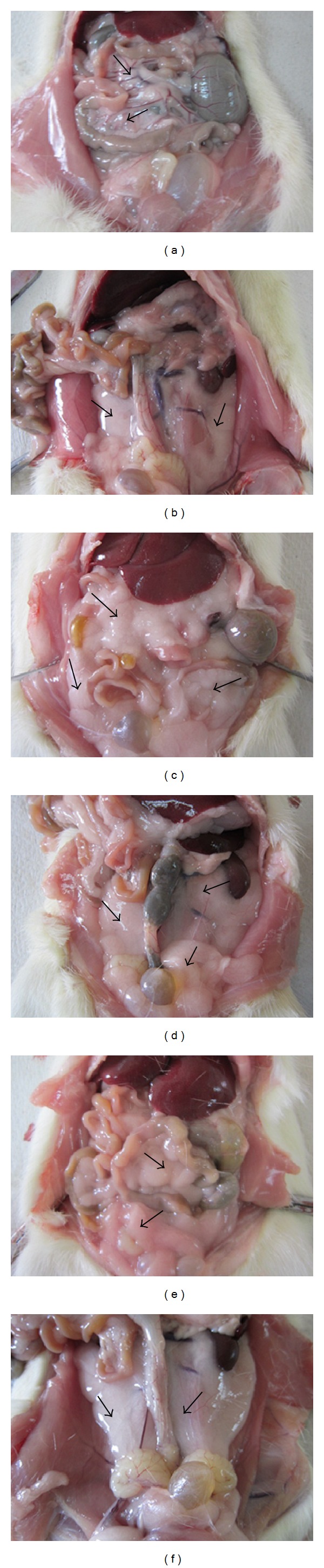
The arrows show the depositions of abdominal adipose tissue which include the mesentery, epididymal, and retroperitoneal for C (a, b), F20 (c, d), and F25 (e, f) groups. The depositions of abdominal adipose tissue were greater in the F20 (c, d) and F25 (e, f) groups as compared to C group (a, b) after 8 weeks of consumption of FDW.

The higher total calorie intake in the F20 group leads to significantly higher percentage in body weight gain, BMI, and AC compared to F25 group over a period of eight weeks (Table 2). The* in situ* observation showed deposition of abdominal adipose tissue as well as the weight of total abdominal adipose tissue deposition which included the mesentery, retroperitoneal, and epididymal fat, which was also significantly higher in F20 group compared to the F25 group (Figure 1).

### 3.3. Blood Pressure

Both groups F20 and F25 showed significantly higher level of systolic blood pressure following consumption of FDW 20% and 25% compared to the C group (Table 2). Although, the differences in systolic blood pressure were not significantly different between F20 and F25 groups, the animals which consumed the most calories developed the highest systolic blood pressure.

### 3.4. Blood Biochemistry

Serum TG levels differed significantly in the F20 and F25 groups compared to C group (Table 2). The group that were given access to FDW 20% had greater TG level than the group given access to FDW 25%. But this difference was not statistically significant. On the other hand, no significant difference was measured in the TC level at the end of the experiment.

Both concentrations of FDW 20% and FDW 25% resulted in significantly higher fasting plasma glucose compared to C group. Though the F25 group showed less fluid intake but it resulted in numerically higher level of plasma glucose, in contrast to F20. However, there was no significant difference between these groups (Table 2).

### 3.5. Gross and Microscopic Changes of Adipose Tissue

**Figure 2:**
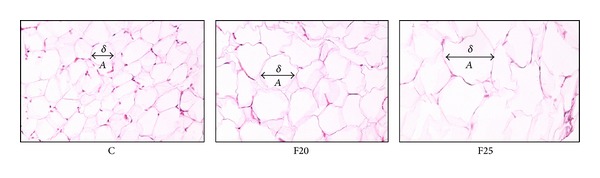
The histomorphology of adipocytes of C group is different as compared to F20 and F25 where the size of adipocytes is increased in F20 and F25 as compared to C group. The size of adipocyte which is indicated by diameter, perimeter, and area and the number of adipocytes also was calculated. *A*: adipocyte, *δ*: diameter.

**Figure 3:**
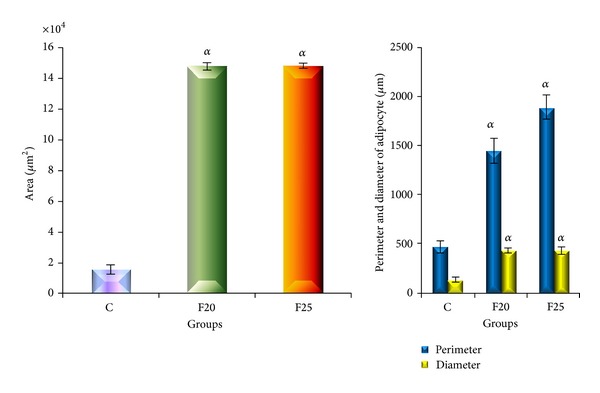
The size of adipocytes was indicated by area, perimeter, and diameter for C, F20, and F25 groups. Values are mean ± SEM and *n* = 6 for each group. The area, perimeter, and diameter of adipocytes were significantly increased in F20 and F25 groups as compared to C group following consumption of FDW for eight weeks.

**Figure 4:**
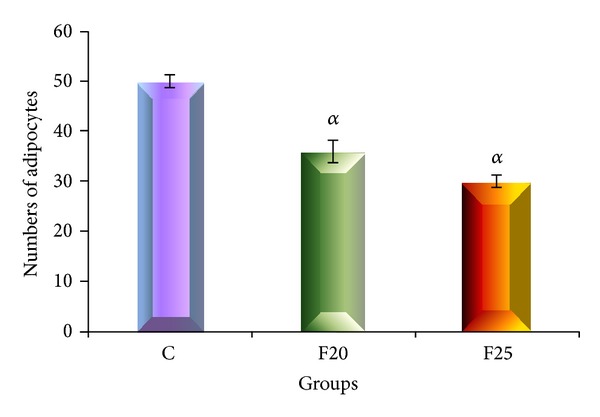
The total number of adipocytes for C, F20, and F25 groups. Values are mean ± SEM and *n* = 6 for each group. Superscript letters are significantly different. There was no increase in the number of adipocytes or hyperplasia in F20 and F25 groups after consumption of FDW for 8 weeks.

Histological findings as shown in Figure 2 revealed hypertrophy of adipocytes in F20 and F25 groups. The size of adipocyte was significantly increased in perimeter, diameter, and area compared to the C group as shown in Figure 3. There was no increase in the number of cells of adipocytes as shown in Figure 4.

## 4. Discussion

Results of the present study showed that fluid intake was the highest in the F20 group compared to the F25 and C groups. This could be attributed to the sweet taste of fructose that enhanced the palatability thereby increasing the fluid intake. However, in F25 group, the fluid intake was far less compared to F20, and it was most probably due to the sweet taste which did not seem to be favourable to the rat. This is an interesting finding because the higher fluid intake and the higher calorie intake would establish the development of metabolic syndrome rat model.

While comparing the consumption of FDW 20% in male SD rats, it was observed that there was no difference in fluid intake when compared to the control group [11]. The striking difference between present study and previous research studies was the type of rat used. In the present study, we used male Wistar rats as this type of rats had higher metabolic rate compared to male SD rats.

The comparison of metabolic effect between Wistar and SD rats was reported previously [10]. Fructose drinking water 10% was administered to both Wistar and SD rats for eight weeks and it showed development of hypertension and hypertriglyceridemia in SD rats. However, no metabolic changes were seen in the Wistar rats [10]. Therefore, for the study of metabolism, the use of Wistar rats is more justified.

To date, metabolic syndrome rat model has been developed using HCHF diet for 16 weeks which include the source of carbohydrate from the FDW 25% in male Wistar rats [12]. At eight weeks, the physiological and metabolic parameters were measured and it revealed that the fluid intake was 18 mL/day. This clearly showed that the amount of fluid intake was reduced compared to our study which was 60.80 mL/day for F20 and 40.52 mL/day for F25 group, which suggested that HCHF diet causes stimulation of satiety centre thereby reducing the fluid intake.

Consumption of FDW causes reduction in the amount of food intake. These results were consistent with the previous studies. Even though the amount of food intake was lower in F20 and F25 group, the total calorie intake was higher in these groups compared to C group. Obviously, this calorie intake was contributed by the calorie from the FDW. 1 g of standard rat chow contributes to 2.8 Kcal. Meanwhile, 1 g of fructose yields 4 Kcal. Again, this study was consistent with past studies.

The results of the present study also showed that F20 group had higher calorie intake compared to the F25 group. With increase in calorie intake, there is more deposition of lipid, which contributes to the development of obesity and metabolic syndrome.

Three indicators of obesity including the percentage of body weight gain, BMI, and AC were measured and were higher in F20 group compared to F25 group. The normal BMI for male Wistar rat was in the range of 0.45 ± 0.02 g/cm² to 0.68 ± 0.05 g/cm² [15]. Our study showed that, following eight weeks of consumption of FDW, F20 group had higher BMI compared to the F25 which was 0.91 g/cm² compared to 0.77 g/cm².

Abdominal circumference also showed higher values in F20 compared to the F25 group. These results showed that the consumption of FDW 20% resulted in higher calorie intake. Hence, there was more deposition of lipid [19].

Depositions of abdominal adipose tissue and its weight also were higher in F20 group. The increment of calorie intake causes the excessive energy to be stored as triglyceride (TG). It then is stored in the adipose tissue and acts as an energy reservoir [20]. The depositions of abdominal adipose tissue are important components in the development of dyslipidemia, hyperglycemia, and hypertension [21]. Adipose tissue acts as an endocrine organ which involves the metabolism of glucocorticoid which is a steroid hormone. Dysregulation of glucocorticoid metabolism leads to obesity, dyslipidemia, hypertension, and diabetes [21]. The depositions of adipose tissue in the abdominal region lead to the formation of obesity central.

In obese state, this adipose tissue tends to be enlarged [20]. Adipocyte is the main cell present in the adipose tissue [20]. In obesity, the cells undergo two processes which are hypertrophy and hyperplasia. The cells first undergo the hypertrophy process and once they reach the maximum size, they undergo hyperplasia process [22].

The present study showed that with eight weeks consumption of FDW, hypertrophy of adipocytes occurred without hyperplasia. We hypothesized with prolongation of the duration of the experiment the cells may undergo hyperplastic changes.

Systolic hypertension also was able to develop with the consumption of FDW in both groups F20 and F25. The mechanisms involved in the formation of SBP in obesity state were due to the increase in cardiac output (CO) and total peripheral resistance (TPR). The sympathetic system and renin-angiotensin system (RAS) are activated in obese and lead to the increment of CO [23]. These factors were proven when the RAS and sympathetic system were inhibited pharmacologically, and the blood pressure reduced to about 50% to 60% [24]. Apart from that, sympathetic activity together with the compression of kidney by the adipose tissue around it causes activation of the RAS [25]. The activation of RAS causes retention of sodium and water by angiotensinogen and leads to the development of hypertension [25].

Excessive formation of TG in the blood is known as hypertriglyceridemia. The insulin sensitivity is reduced with the presence of TG [26]. Thus, it results in reduction of glucose uptake by the insulin sensitive tissue. This further leads to ongoing lipolysis process and more free fatty acids and glycerol formed [26]. The FFA and glycerol then enter the adipose tissue to form TG [26]. These viscous cycles are repeated and more TG is formed.

The results of present study showed that the formation of hypertriglyceridemia occurs following eight weeks of consumption of FDW with the value of 1.22 mmol/L and 1.13 mmol/L for F20 and F25 groups each. Meanwhile, the development of metabolic syndrome with HCHF diet for eight weeks showed the level of TG to be 0.3 mmol/L [12].

Hyperglycemic state was achieved with consumption of FDW in F20 and F25 groups. This achievement is due to the fact that, unlike glucose, fructose does not stimulate the secretion of insulin from pancreatic *β*-cell [7]. Furthermore, the reduction of insulin sensitivity in the state of hypertriglyceridemia may lead to the formation of hyperglycemia.

## 5. Conclusion

Although past research developed metabolic syndrome rat model by induction of HCHF diet for 16 weeks, we performed a new metabolic syndrome rat model in an easier way which was cost-effective and had a shorter duration of induction which was by manipulation of drinking water even with the normal food consumption for eight weeks. Although metabolic syndrome criteria can be achieved by FDW 20% and 25%, the FDW 20% proved to be easily drunk by the rats. Hence, the total calorie intake was significantly greater than that of FDW 25% which posed the striking difference between these two concentrations. This leads to higher obesity parameter which was the cause of the development of metabolic syndrome. We conclude that the metabolic syndrome rat model is best established with the induction of FDW 20% for eight weeks in male Wistar rat as it caused full blown metabolic syndrome in all parameters with higher obesity parameter compared to FDW 25%.

## Figures and Tables

**Table 1:** Effects of fructose drinking water on physiological variables in C, F20, and F25 groups for 8 weeks.

Variables	C	F20	F25
Food intake (g/day)	23.80 ± 1.20	13.87 ± 0.73^a^	13.82 ± 0.22^a^
Fluid intake (mL/day)	53.54 ± 0.78	60.80 ± 1.66^ab^	40.52 ± 1.84^ad^
Total calorie intake (Kcal/day)	62.98 ± 3.16	102.70 ± 3.43^ab^	84.32 ± 2.27^ad^

Values are mean ± SEM and  *n* = 6  for each group. Superscript letters are significantly different. ^a^ *P* < 0.05 indicates a significant difference as compared to C group. ^b^ *P* < 0.05 indicates a significant difference as compared to F25 group. ^d^ *P* < 0.05 indicates a significant difference as compared to F20 group.

**Table 2:** Effects of fructose drinking water on metabolic variables in C, F20, and F25 groups for 8 weeks.

Variables	C		F20		F25	
	Baseline	8 weeks	Baseline	8 weeks	Baseline	8 weeks
Body weight gain (%)	—	36.12 ± 0.81	—	43.03 ± 0.76^ab^	—	39.75 ± 0.33^ad^
Body mass index (g/cm²)	0.64 ± 0.01	0.66 ± 0.02	0.64 ± 0.02	0.91 ± 0.02^abc^	0.66 ± 0.01	0.77 ± 0.01^abd^
Abdominal circumference (cm)	16.5 ± 0.26	18.3 ± 0.1	16.1 ± 0.40	22.9 ± 0.3^abc^	16.5 ± 0.32	21.1 ± 0.4^abd^
Total abdominal fat (mg/mm tibial length)	—	196.72 ± 23.13	—	437.97 ± 27.08^ac^	—	347.48 ± 22.66^ad^
Plasma triglyceride (mmol/L)	0.70 ± 0.09	0.65 ± 0.08	0.78 ± 0.07	1.22 ± 0.14^ab^	0.70 ± 0.08	1.13 ± 0.09^ab^
Plasma total cholesterol (mmol/L)	1.5 ± 0.01	1.6 ± 0.1	1.5 ± 0.04	1.5 ± 0.1	1.5 ± 0.11	1.5 ± 0.1
Systolic blood pressure (mmHg)	103.3 ± 1.1	105.0 ± 1.8	101.3 ± 2.1	145.8 ± 1.5^ab^	104.5 ± 1.1	142.5 ± 1.1^ab^
Plasma glucose (mmol/L)	5.1 ± 0.4	6.4 ± 0.2	4.7 ± 0.4	8.1 ± 0.6^ab^	4.8 ± 0.5	8.4 ± 0.9^ab^

Values are mean ± SEM and  *n* = 6 for each group. Superscript letters are significantly different. ^a^A significant difference as compared to C group at 8 weeks. ^b^A significant difference within group as compared to baseline. ^c^A significant difference as compared to F25 group at 8 weeks. ^d^A significant difference as compared to F20 group at 8 weeks.
